# Plasma MicroRNA Signature of Alcohol Consumption: The Rotterdam Study

**DOI:** 10.1093/jn/nxac216

**Published:** 2022-09-20

**Authors:** Irma Karabegović, Yasir Abozaid, Silvana C E Maas, Jeremy Labrecque, Daniel Bos, Robert J De Knegt, M Arfan Ikram, Trudy Voortman, Mohsen Ghanbari

**Affiliations:** Department of Epidemiology, Erasmus MC University Medical Center, Rotterdam, The Netherlands; Department of Epidemiology, Erasmus MC University Medical Center, Rotterdam, The Netherlands; Department of Epidemiology, Erasmus MC University Medical Center, Rotterdam, The Netherlands; Vall d'Hebron Institute of Oncology (VHIO), Barcelona, Spain; Department of Epidemiology, Erasmus MC University Medical Center, Rotterdam, The Netherlands; Department of Epidemiology, Erasmus MC University Medical Center, Rotterdam, The Netherlands; Department of Radiology and Nuclear Medicine, Erasmus MC University Medical Center, Rotterdam, The Netherlands; Department of Gastroenterology, Erasmus MC University Medical Center, Rotterdam, The Netherlands; Department of Epidemiology, Erasmus MC University Medical Center, Rotterdam, The Netherlands; Department of Epidemiology, Erasmus MC University Medical Center, Rotterdam, The Netherlands; Division of Human Nutrition and Health, Wageningen University & Research, Wageningen, The Netherlands; Department of Epidemiology, Erasmus MC University Medical Center, Rotterdam, The Netherlands

**Keywords:** plasma miRNAs, alcohol consumption, liver disease, miR-3937, miR-122-5p

## Abstract

**Background:**

MicroRNAs (miRNAs) represent a class of noncoding RNAs that regulate gene expression and are implicated in the pathogenesis of different diseases. Alcohol consumption might affect the expression of miRNAs, which in turn could play a role in risk of diseases.

**Objectives:**

We investigated whether plasma concentrations of miRNAs are altered by alcohol consumption. Given the existing evidence showing the link between alcohol and liver diseases, we further explored the extent to which these associations are mediated by miRNAs.

**Methods:**

Profiling of plasma miRNAs was conducted using the HTG EdgeSeq miRNA Whole Transcriptome Assay in 1933 participants of the Rotterdam Study. Linear regression was implemented to explore the link between alcohol consumption (glasses/d) and miRNA concentrations, adjusted for age, sex, cohort, BMI, and smoking. Sensitivity analysis for alcohol categories (nondrinkers, light drinkers, and heavy drinkers) was performed, where light drinkers corresponded to 0–2 glasses/d in men and 0–1 glasses/d in women, and heavy drinkers to >2 glasses/d in men and >1 glass/d in women. Moreover, we utilized the alcohol-associated miRNAs to explore their potential mediatory role between alcohol consumption and liver-related traits. Finally, we retrieved putative target genes of identified miRNAs to gain an understanding of the molecular pathways concerning alcohol consumption.

**Results:**

Plasma concentrations of miR-193b-3p, miR-122-5p, miR-3937, and miR-4507 were significantly associated with alcohol consumption surpassing the Bonferroni-corrected *P* < 8.46 × 10^−5^. The top significant association was observed for miR-193b-3p (β = 0.087, *P* = 2.90 × 10^−5^). Furthermore, a potential mediatory role of miR-3937 and miR-122-5p was observed between alcohol consumption and liver traits. Pathway analysis of putative target genes revealed involvement in biological regulation and cellular processes.

**Conclusions:**

This study indicates that alcohol consumption is associated with plasma concentrations of 4 miRNAs. We outline a potential mediatory role of 2 alcohol-associated miRNAs (miR-3937 and miR-122-5p), laying the groundwork for further exploration of miRNAs as potential mediators between lifestyle factors and disease development.

## Introduction

Alcohol consumption is a modifiable lifestyle factor and a leading risk factor for the global burden of many diseases. Given its widespread nature, alcohol has been estimated to contribute to 2.7 million deaths and 4% of the global disease burden annually (1). High alcohol intake has been associated with an increased risk of stroke, peripheral artery disease (2), liver diseases (3–6), various cancers (7–10), overall all-cause mortality (11), and many other diseases (12). Although numerous molecular mechanisms have been postulated to explain the link between alcohol consumption and the risk of various diseases, this complex etiology remains to be explored (13–15). The liver is the primary organ for metabolizing and detoxification of alcohol (16), while excessive alcohol consumption can have a severe impact on liver health, including fatty liver, alcoholic hepatitis, and cirrhosis (17). In addition, only 10%–20% of chronic alcohol consumers will progress to advanced alcoholic liver disease (18). The exact molecular mechanisms involved in alcohol-related liver traits and diseases are still not fully elucidated (19, 20). Behavioral factors, including alcohol consumption, have been linked with epigenetic markers (21–23), and these epigenetic markers have also been linked to several diseases (20). Epigenetic mechanisms include DNA methylation, histone protein modifications, and RNA-mediated regulation by noncoding RNAs (20, 24).

MicroRNAs (miRNAs) are small noncoding RNA molecules (∼22 nucleotides in length) that regulate gene expression at the posttranscriptional level. As such, miRNAs are estimated to regulate the expression of more than half of the protein-coding genes in our genome (25). They are considered as a type of epigenetic regulation whose mechanism of action relies on the degradation of mRNAs and translational repression (26). An extensive body of research has demonstrated that dysregulation of miRNAs is associated with disease risk (27–32). Moreover, recent studies have indicated an influence of modifiable lifestyle factors (such as smoking and diet) on miRNA expression levels (33). Two before–after studies with small sample sizes (*n* = 16–18) (34, 35) showed differential expression of miRNAs after exposure to alcohol consumption, including miR-122-5p, a highly expressed liver miRNA. However, limited studies were conducted to explore the association between expression levels of miRNAs and alcohol consumption in larger sample sizes (33). Because identifying alcohol-associated changes in miRNA expression might help to elucidate the mechanism of action between alcohol consumption and health outcomes, it is of crucial importance to explore this niche. In this study, we aimed to investigate the association of plasma miRNAs with alcohol consumption and to explore whether there is a mediating effect for the alcohol-associated miRNAs in the cross-sectional association of alcohol consumption with liver function and disease, using data from the large population-based prospective Rotterdam Study (RS) cohort (36).

## Methods

### Study population

This study was conducted in the RS, which is an ongoing prospective population-based cohort study. In brief, the RS consists of 4 subcohorts. The first subcohort (RS-I) was initiated in 1990 with individuals ≥55 y of age (*n* = 7983). The study was extended by including a second subcohort (RS-II) in 2000 (*n* = 3011, ≥55 y of age), a third subcohort (RS-III) in 2006 (*n* = 3932, ≥45 y of age), and the most recent fourth subcohort (RS-IV) in 2016 (*n* = 3005, ≥40 y of age). In addition to these baseline examinations, the participants were re-examined during follow-up every 3–5 y. More in-depth details regarding the design of the RS can be found elsewhere (36).

For the present study, 1000 participants were included from the fourth visit of RS-I (RS-I-4) and 1000 participants from the second visit of RS-II (RS-II-2), for whom we had miRNA expression data measured (total *n* = 2000). These visits of the RS occurred between 2002 and 2005. From the 2000 unique individuals, 1 participant was excluded owing to missing profiling data for all miRNAs, whereas 66 were excluded owing to missing data on alcohol consumption. In total, 1933 nonoverlapping participants were included in our analysis. The RS has been approved by the Medical Ethics Committee of the Erasmus Medical Center and by the Dutch Ministry of Health, Welfare, and Sport (36).

### miRNA expression profiling

Blood samples were collected in EDTA-treated containers , followed by separation of plasma into aliquots and storage at −80°C, according to standard procedures. Plasma samples were then used for miRNA expression profiling using the HTG EdgeSeq miRNA Whole Transcriptome Assay (WTA) (HTG Molecular Diagnostics). The WTA measured the expression of 2083 human miRNAs using the Illumina NextSeq sequencer (Illumina). The assay characterizes miRNA expression patterns and hereby measured the expression of 13 housekeeping genes, providing flexibility in data analysis and normalization. The miRNA expression quantification was based on counts per million (CPM), which were log2 transformed and used as standardization, adjusting for total reads within each sample. Furthermore, the miRNAs showing log2 CPM < 1.0 were referred to as low expressed; whereas, the well-expressed miRNAs were defined as those with >50% of values above the lower limit of quantification (LLOQ), resulting in a total of 591 miRNAs, which were used in our analysis. The LLOQ was used for the selection of well-expressed miRNAs (*n* = 591), which was based on a monotonic decreasing spline curve fit between the means and SDs of all miRNAs in the whole sample of study participants.

### Assessment of alcohol consumption

Participants were administered interviews at home by research assistants, where they were asked about their alcohol consumption. The first question asked whether participants ever drank alcohol. If the answer was confirmative, it was later followed by more extensive questions on the type of alcohol (e.g., beer; red wine; white wine; moderately strong spirits such as Campari, Martini, and sherry; and strong spirits such as rum, brandy, and whisky) and frequency of consumption per week. This information was collected and used to calculate the average alcohol consumption in glasses per day. The glasses per day information could be used to estimate grams of alcohol, assuming that 1 glass of alcohol would roughly correspond to 10 g of alcohol (37). Because our study population also included a percentage of alcohol nondrinkers (*n* = 307, 15.88%), the alcohol consumption variable was right-skewed. To satisfy the assumption of normality of residuals in linear regression, we applied transformation of [log(glasses/d + 1)], according to the approach reported by Liu et al. (38). Furthermore, alcohol consumption was categorized into nondrinkers (0 glasses/d), light drinkers (0–2 glasses/d in men and 0–1 glasses/d in women), and heavy drinkers (>2 glasses/d in men and >1 glass/d in women).

### Assessment of covariates

Questionnaires were used to assess the participants’ age, sex, and smoking status (classified as current, former, and never smokers). Furthermore, the height and weight of participants were measured with the participants standing without heavy garments or shoes. BMI was computed as weight in kilograms divided by height in meters squared (kg/m^2^).

### Assessment of fatty liver and hepatic steatosis using computed tomography (CT) scan and ultrasound

A multidetector CT scanner (Somatom Sensation 16 or 64, Siemens) was acquired as part of a larger project on vascular calcification. For the current project, the Electorcardiogram-gated, noncontrast cardiac scan was used to assess the density of the liver, as a proxy for fatty liver disease. Detailed imaging parameters are described in detail elsewhere (39). We assessed the density of the liver using a standardized strategy that included drawing 3 circular regions of interest (ROIs) in liver tissue in which the mean liver attenuation (LA) was calculated (40). The ROIs were carefully chosen to include solely liver tissue (avoiding disruptive tissue such as focal lesions, cysts, or large blood vessels). Next, we determined the mean Hounsfield unit (HU) value from the retrieved 3 measurements as an indicator of the total liver fat amount. As the amount of liver fat is increased, the measured LA is decreased; therefore, a lower LA indicates a higher risk of fatty liver. All measurements were computed using Philips iSite Enterprise software (Royal Philips Electronics NV 2006), described in depth elsewhere (41). In addition, we transformed liver fat (A) using exponential values (B) (B = A^3.5^/10,000) because it was left-skewed (41).

Beyond the CT assessment, hepatic steatosis was determined by using abdominal ultrasound (US) data, generated via a Hitachi HI VISION 900 by an experienced and certified technician (**Supplemental Table 1**). Steatosis was diagnosed by dichotomizing the data into the presence or absence of hyperechogenic liver parenchyma, as reported previously (42). More details on liver steatosis and nonalcoholic fatty liver disease (NAFLD) within the RS can be found elsewhere (43).

### Measuring liver enzymes

Serum γ-glutamyl transferase (GGT) and alkaline phosphatase (ALP) concentrations were determined within 2 wk of collecting and stored with nonfasting and fasting blood samples at −20°C. The Merck Diagnostica (Merck) kit was used on an Elan Autoanalyzer (Merck). Furthermore, considering local cutoffs, elevated GGT was defined as >34 U/L for women and >49 U/L for men, whereas ALP was considered elevated at >97 U/L for women and >114 U/L for men; more details can be found elsewhere (44). To satisfy the assumption of normality of residuals, because GGT and ALP were right-skewed, we applied log transformation.

### Statistical analyses

#### Alcohol consumption in association with alterations in miRNA concentrations

**FIGURE 1:**
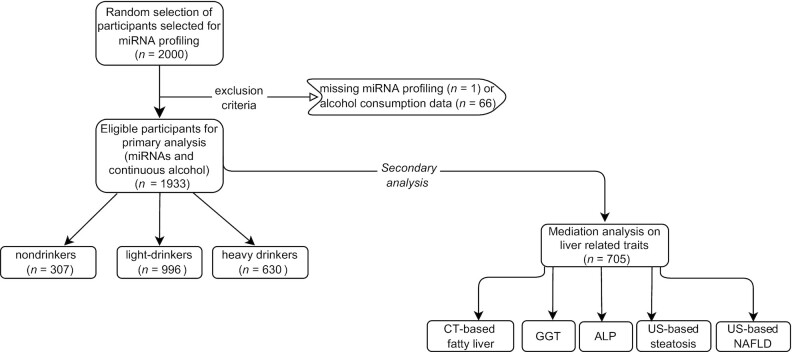
Overview of the study design. The flowchart summarizes the sample sizes for the different analyses. The main analysis investigating the association between alcohol consumption and miRNA expression was performed on participants from RS-I-4 and RS-II-2 within the RS, who had data available on miRNA concentrations and alcohol consumption (*n* = 1933). Nondrinkers: 0 glasses/d; light drinkers: 0–2 glasses/d in men and 0–1 glasses/d in women; heavy drinkers: >2 glasses/d in men and >1 glass/d in women. ALP, alkaline phosphatase; CT, computed tomography; GGT, γ-glutamyl transferase; miRNA, microRNA; NAFLD, nonalcoholic fatty liver disease; RS, Rotterdam Study; US, ultrasound.

Multivariable linear regression models were implemented to explore the association between alcohol consumption as the main exposure [log(glasses/d + 1)] and plasma miRNA concentrations (log2 CPM) as the outcome. For a more detailed overview of the inclusion criteria and the analysis workflow, see **Figure 1**. We tested 3 different models. The first model was adjusted for age, sex, and cohort; the second model was further adjusted for BMI; whereas in the final model we additionally adjusted for smoking status. The main results were reported from the fully adjusted model. The Bonferroni-corrected *P* value threshold < 0.05/591 = 8.46 × 10^−5^ (after adjustment based on the number of miRNAs tested) was set for our hypothesis-free approach. The assumptions of linear regression analysis including normality of residuals, normality of random effects, multicollinearity, linear relation, and homogeneity of variance were assessed using the “performance” package in R.

Furthermore, for the alcohol-associated miRNAs, we performed a sensitivity analysis, where we treated alcohol exposure as a categoric variable. The nondrinker category was included as the reference group, where it was compared with the light and heavy drinkers.

Moreover, because alcohol consumption might have sex-specific differences due to differential drinking patterns (45) or alcohol metabolism (46), we performed a sex-stratified analysis to explore potential changes in alcohol-associated miRNAs.

#### Mediation analyses with liver traits

In our secondary objective, we performed mediation analyses, where our exposure was always alcohol consumption, the mediators were miRNAs associated with both alcohol consumption and liver disease, and the outcomes were liver-related traits, including CT-based LA, liver enzymes (GGT and ALP), US-based hepatic steatosis, and NAFLD. For the continuous outcomes (CT-based LA, GGT, and ALP) we used linear regression, whereas for binary outcomes (steatosis and NAFLD) we used logistic regression. The selection criteria of potential mediators were based on a seminal article by Baron and Kenny (47), stating that in order to define a variable as a mediator, there should be a significant relation between the mediator (miRNAs) and the outcome (liver-related traits). In that line, 3 of the alcohol-associated miRNAs (miR-193b-3p, miR-122-5p, and miR-3937) were previously associated with liver-related traits within the RS (48), hence they were included as mediators in our analyses. Our mediation analyses were implemented using 2-way decomposition assessing the direct and indirect effects, meaning that the overall effect of alcohol consumption on liver-related traits with miRNAs as mediators was decomposed into 2 main components: *1*) the direct effect of alcohol consumption on liver-related traits (i.e., LA, GGT, ALP, steatosis, and NAFLD) in the absence of mediators (i.e., miR-193b-3p, miR-122-5p, or miR-3937) and *2*) the indirect effect. Models were adjusted for the same confounders as in the main analysis, including age, sex, cohort, BMI, and smoking status. In addition, we also assessed if there was a potential interaction effect between the exposure and the mediator. For the models that showed the presence of an interaction effect (*P* < 0.05), we implemented exposure and mediator interaction terms in mediation analyses.  All the confounders included in the statistical analyses were obtained at the same time point as the miRNA expression data, as well as the data on CT-based LA and liver enzymes (RS-I-4 and RS-II-2), whereas the data based on US (steatosis and NAFLD) were collected during a follow-up visit and analyzed in the longitudinal setting. We used the “mediate” function from the mediation package (49) to obtain the average causal mediation effect (ACME), average direct effect, total effect, and proportion mediated per model. Mediation results were based on quasi-Bayesian approximation with 1000 simulations.

Furthermore, the mediation analyses performed assumed no unmeasured confounding. As such, we included bias analyses using the “medsens” function from the mediation package (49) to determine the ρ at which ACME was 0 per model. A value of ρ close to 0 reflects that the assumption of no additional unmeasured confounding is sensitive to violations and likely does not hold. We implemented recommended AGReMa Statement guidelines when reporting the results (50), including reporting baseline characteristics as well as potential confounders in Supplemental Table 1.

#### Mendelian randomization

We investigated the causal relation between the alcohol-associated miRNAs and liver-related traits by utilizing the 2-sample Mendelian randomization (MR) approach. Instrumental variables (IVs) for each of the alcohol-associated miRNAs were extracted using different resources, including a genome-wide association study (GWAS) conducted in the RS (*n* = 1687) (data not shown) and publicly available GWASs on miRNAs (51–53). We identified 10 cis-miRNA expression quantitative trait loci (miR-eQTL)s for miR-193b-3p (53), whereas miR-193b-3p and miR-122-5p only had trans-miR-eQTLs (51). The trans-eQTLs were excluded from our further analysis owing to the assumption of no horizontal pleiotropy (53, 54). Next, the cis-miR-eQTLs of miR-193b-3p were pruned at *R*^2^ < 0.01, to remove correlated single-nucleotide polymorphisms (SNPs). This left us with a single SNP (rs30227) to be used as an IV. IVs on liver traits were extracted from the IEU GWAS database release (https://gwas.mrcieu.ac.uk/), where we included the following traits: liver fat percentage (55), NAFLD (https://finngen.gitbook.io/documentation/), and liver enzymes (56). MR was performed using the “TwoSampleMR” package in R, by implementing the Wald ratio because a single SNP was available to be used as an IV.

Our analyses were performed using R software, version 4.1.1 (R Core Team, 2021). Moreover we used the following packages for different utilities within R: rio (version 0.5.27) (57) for data importing/exporting; tidyverse (version 1.3.1) (58), janitor (version 2.1.0) (59), and lubridate (version 1.7.10) (60) for data manipulation and handling; stats (version 4.1.1) (61), broom (version 0.7.9) (62), performance (version 0.9.1) (63), and purrr (version 0.3.4) (64) for modeling; ggplot2 (version 3.3.5) (65) for visualization; mediation (version 4.5.0) (49) for mediation analyses; TwoSampleMR (version 0.5.6) for MR analysis (66); and tableone (version 0.13.0) (67) for clinical characteristics.

#### In silico analyses of alcohol-associated miRNAs

We explored if the alcohol-associated miRNAs are expressed in the liver by using the Human miRNA tissue atlas (https://ccb-web.cs.uni-saarland.de/tissueatlas) (68, 69). More details regarding the tissue specificity index (TSI) can be found elsewhere (69). As an additional analysis, we utilized 3 universally used miRNA target gene prediction databases: TargetScan (70), miRTarBase (71), and miRDB (72), to identify putative target genes of the alcohol-associated miRNAs. Applying a cutoff based on a total context score of ≤ −0.60, we selected target genes using TargetScan, whereas for miRDB we applied selection on target scores ≥ 60. The scores of the 2 databases are explained in detail elsewhere (70, 73). In addition, we used miRTarBase (71) to select the target genes that were proven by experimental validation methods, such as reporter assay, qPCR, and western blot. We focused on genes that were available in any 2 out of the 3 aforementioned databases. Furthermore, we investigated if any of these predicted target genes had been associated previously with alcohol consumption and/or alcohol use disorder by either a review, a GWAS, an epigenome-wide association study (EWAS), or a transcriptome-wide association study on alcohol consumption (33, 38, 74, 75). Finally, the putative target genes we obtained from the analysis described already were used for gene ontology analysis to explore the biological processes these genes might be involved in (76), by utilizing the publicly available Web tool PANTHER (http://www.pantherdb.org/) (77).

## Results

**Table 1** presents characteristics of the study population (*n* = 1933). The mean ± SD age of the study population was 71.62 ± 7.5 y, with a BMI of 27.65 ± 4.13 kg/m^2^ and a median [IQR] alcohol consumption of 0.71 glasses/d [0.07–2.00 glasses/d]. Of the 1933 individuals, 56.8% were women.

**TABLE 1:** Participant characteristics of the study population from RS-I-4 and RS-II-2 within the RS cohort^1^

Variable	*n* = 1933
Age, y	71.62 ± 7.5
Female sex, *n* (%)	1098 (56.8)
BMI, kg/m^2^	27.65 ± 4.13
Smoking	
Current	260 (13.5)
Former	1069 (55.3)
Never	604 (31.2)
Alcohol, glasses/d	0.71 [0.07–2.00]
Nondrinkers	307 (15.9)
Light drinkers	996 (51.5)
Heavy drinkers	630 (32.6)

1 Values are mean ± SD for continuous data and *n* (%) for categoric data, apart from alcohol (glasses/d) which is reported in median [IQR] owing to the distribution of the variable. Alcohol categories were defined as follows: nondrinkers: 0 glasses/d; light drinkers: 0–2 glasses/d in men and 0–1 glasses/d in women; heavy drinkers: >2 glasses/d in men and >1 glass/d in women. RS, Rotterdam Study.

### Plasma miRNAs associated with alcohol consumption

**FIGURE 2:**
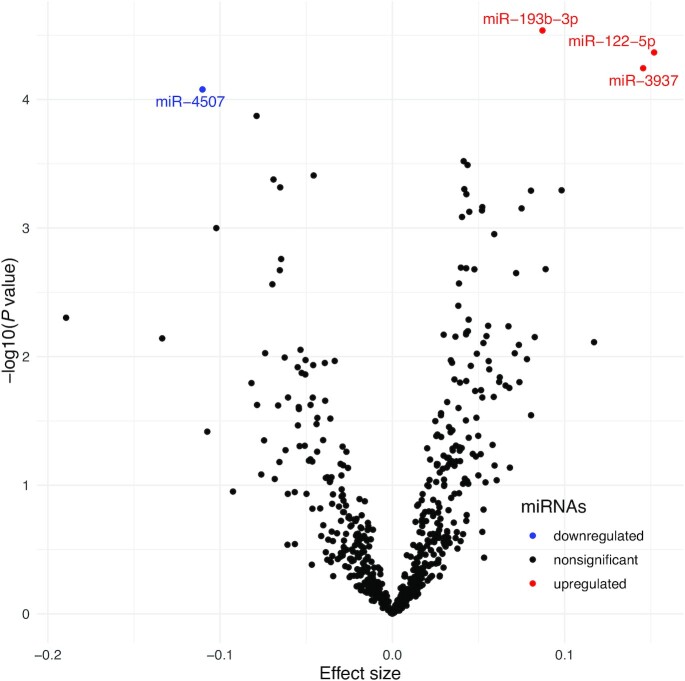
Plasma miRNAs associated with alcohol consumption in glasses per day (*n* = 1933). The volcano plot depicts the measure of effect size against magnitude of significance for the linear regression model testing the association between miRNA expression levels and alcohol consumption, adjusted for age, sex, cohort, BMI, and smoking. The dots indicate each tested miRNA and represent the β coefficients obtained from each linear regression analysis. Red dots indicate positively associated miRNAs, blue dots indicate negatively associated miRNAs, and black dots represent miRNAs that were not significantly associated. miR, microRNA; miRNA, microRNA.

**FIGURE 3:**
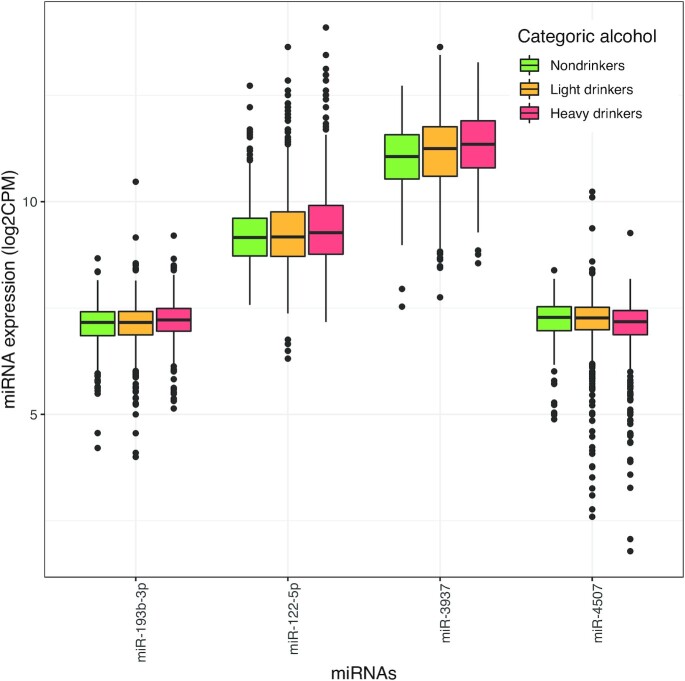
Distribution of the significantly associated miRNAs in the 3 alcohol consumption categories. The *x* axis depicts significantly associated miRNAs with alcohol consumption, the *y* axis depicts miRNA expression levels in log2 CPM, displaying a boxplot of median miRNA expression levels. The horizontal line within each boxplot represents the median, whereas the whiskers depict minimum (corresponding to Q1 − 1.5*IQR) and maximum values (corresponding to Q3 + 1.5*IQR) in the data. Different colors indicate different categories of alcohol consumption: nondrinkers are green (0 glasses/d, *n* = 307), light drinkers are yellow (0–2 glasses/d in men and 0–1 glasses/d in women, *n* = 996), and heavy drinkers are red (>2 glasses/d in men and >1 glass/d in women, *n* = 630). CPM, counts per million; miR, microRNA; miRNA, microRNA.

We found 4 miRNAs to be significantly associated with alcohol consumption (as a continuous variable), surpassing the significance threshold of *P* < 8.5 × 10^−5^. Of these, miR-193b-3p, miR-122-5p, and miR-3937 showed a positive association, whereas miR-4507 was inversely associated with alcohol consumption (**Table 2**, **Figure 2**). Table 2 and **Figure 3** present the results of our sensitivity analysis, where we explored alcohol consumption as a categoric exposure. The categorization of alcohol consumption reduced the power, yet the association of miR-3937 remained statistically significant for heavy drinkers (*P* = 3.02 × 10^−6^) in comparison with nondrinkers. In addition, mean expression of miR-3937 in light drinkers increased by 0.142 compared with the mean of nondrinkers in the reference category, whereas this increase almost doubled (0.273) in heavy drinkers. In contrast, the mean expression of miR-4507 in light drinkers decreased by −0.029 in comparison with the mean of nondrinkers (reference), whereas for heavy drinkers this dropped by −0.155 (Table 2, Figure 3).

**TABLE 2:** Association between miRNAs and alcohol consumption as a continuous variable (glasses/d) and a categoric variable (never drinkers compared with light or heavy drinkers)^1^

	Alcohol, glasses/d (*n* = 1933)			Never drinkers (*n* = 307) vs. light (*n* = 996) or heavy drinkers (*n* = 630)			
miRNA ID	β	SE	*P* value	Category	β	SE	*P* value
miR-193b-3p	0.087	0.020	2.90 × 10^−5^	Light drinkers	0.026	0.031	4.07 × 10^−1^
				Heavy drinkers	0.086	0.033	1.02 × 10^−1^
miR-122-5p	0.151	0.037	4.31 × 10^−5^	Light drinkers	0.015	0.056	7.77 × 10^−1^
				Heavy drinkers	0.125	0.060	3.75 × 10^−2^
miR-3937	0.145	0.036	5.71 × 10^−5^	Light drinkers	0.142	0.054	8.64 × 10^−3^
				Heavy drinkers	0.273	0.058	3.02 × 10^−6^
miR-4507	−0.110	0.027	8.36 × 10^−5^	Light drinkers	−0.029	0.042	4.85 × 10^−1^
				Heavy drinkers	−0.155	0.045	6.26 × 10^−4^

1 On the left side of the table are the results from the linear regression with continuous data on alcohol consumption as the main exposure transformed to [log(glasses/d + 1)], where the analyses were adjusted for age, sex, cohort, BMI, and smoking status. The right side of the table depicts alcohol consumption stratified to a categoric variable (where nondrinkers were treated as a reference) and used as the main exposure for linear regression analysis, adjusted for age, sex, cohort, BMI, and smoking status. In all the analyses presented, miRNA expression levels were outcome variables, and the effect sizes reported are β coefficients from regression analysis. Nondrinkers: 0 glasses/d; light drinkers: 0–2 glasses/d in men and 0–1 glasses/d in women; heavy drinkers: >2 glasses/d in men and >1 glass/d in women. miR, microRNA; miRNA, microRNA.

Furthermore, in the sex-stratified analysis, we observed that all the effect size estimates were in the same direction. However, most of the alcohol-associated miRNAs had stronger effect size estimates in men, except for miR-4507 which showed more decrease in women than in men (**Supplemental Table 2**).

### Mediation analyses for alcohol consumption, miRNA expression, and liver disease

**FIGURE 4:**
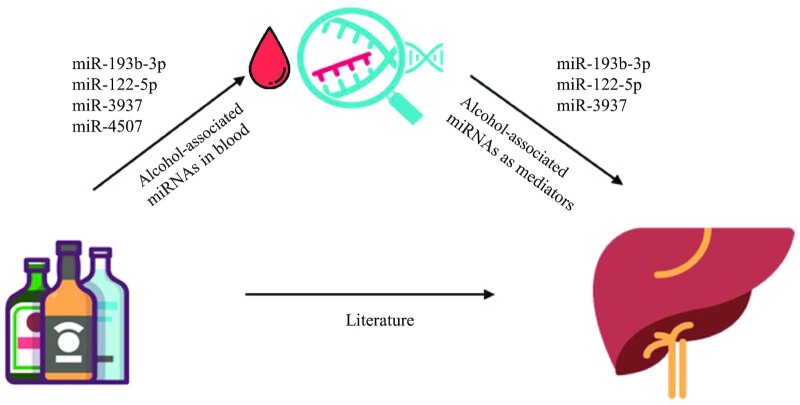
Conceptual diagram showing the relation between alcohol consumption and liver health, and the potential mediatory role of alcohol-associated miRNAs. The conceptual diagram depicts the relation between exposure (alcohol consumption), outcome [liver-related traits including CT-based liver attenuation, liver enzymes (γ-glutamyl transferase and alkaline phosphatase), and ultrasound-based hepatic steatosis and nonalcoholic fatty liver disease], and the mediators (miRNA expression level). CT, computed tomography; miRNA, microRNA.

We tested the potential mediatory role of 3 miRNAs previously shown to be associated with fatty liver disease (miR-193b-3p, miR-122-5p, and miR-3937) (47, 48) in the association between alcohol and liver function and disease (**Figure 4**). Supplemental Table 1 presents the descriptive characteristics of this subset of participants (*n* = 705). We performed mediated interaction terms for all the models, of which 1 model suggested an interaction effect between mediator and exposure: miR-122-5p and alcohol on ALP (*P* = 0.04) (**Supplemental Table 3)**. For this model, we included interaction terms in the main analysis, whereas for the other models we did not include any interaction terms (**Table 3**). Out of all the mediation analyses performed, we identified a mediatory role of miR-3937 in the association between alcohol and CT-based fatty liver as well as GGT, whereas miR-122-5p showed a mediatory role between alcohol and CT-based fatty liver disease, GGT, and US-based steatosis (Table 3). We performed the bias analysis testing violation of the assumption of no unmeasured confounding in the mediation analyses. We conducted ρ at which ACME was 0 and obtained ρ values in the range between −0.1 and 0.4 (Table 3). A value of ρ close to 0 indicates that the assumption of no unmeasured confounding was sensitive to violation.

**TABLE 3:** Mediation analysis of 3 alcohol-associated miRNAs with alcohol consumption and liver-related traits [CT-based liver attenuation, liver enzymes (GGT and ALP), and US-based hepatic steatosis and NAFLD] in Rotterdam Study participants^1^

miRNA ID	Liver-related traits (*n* = 705)	ACME (95% CI)	ADE (95% CI)	Total effect (95% CI)	Prop. Med. (95% CI)	${\rm{\rho }}$ at which ACME is 0
miR-3937	CT-based fatty liver	1.630 (0.114, 3.490)	−24.80 (−34.72, −15.66)	−23.17 (−33.25, −14.14)	−0.070 (−0.187, −0.004)	0.1
	GGT	−0.009 (−0.024, −0.0001)	0.287 (0.209, 0.375)	0.277 (0.199, 0.362)	−0.035 (−0.094, −0.0006)	−0.1
	ALP	−0.003 (−0.009, 0.00005)	−0.028 (−0.065, 0.009)	−0.031 (−0.068, 0.005)	0.117 (−0.351, 0.852)	−0.1
	US-based steatosis	−0.006 (−0.017, −0.0001)	0.063 (−0.0009, 0.127)	0.057 (−0.008, 0.121)	−0.108 (−0.779, 0.454)	−0.1
	US-based NAFLD	−0.004 (−0.014, 0.0006)	0.255 (0.189, 0.317)	0.251 (0.184, 0.310)	−0.019 (−0.061, 0.002)	−0.1
miR-122-5p	CT-based fatty liver	−1.394 (−3.115, −0.194)	−21.77 (−31.39, −10.92)	−23.17 (−32.77, −11.88)	0.060 (0.007, 0.166)	−0.1
	GGT	0.036 (0.004, 0.071)	0.241 (0.166, 0.314)	0.277 (0.197, 0.360)	0.131 (0.017, 0.238)	0.4
	ALP	0.002 (−0.0008, 0.006)	−0.032 (−0.069, 0.009)	−0.028 (−0.067, 0.073)	−0.080 (−0.875, 0.644)	0
	US-based steatosis	0.008 (0.001, 0.020)	0.047 (−0.016, 0.109)	0.056 (−0.006, 0.117)	0.155 (−0.702, 1.188)	0.1
	US-based NAFLD	0.005 (−0.0001, 0.014)	0.244 (0.175, 0.305)	0.250 (0.183, 0.310)	0.022 (0.000, 0.062)	0.1
miR-193b-3p	CT-based fatty liver	−1.116 (−2.842, 0.344)	−22.05 (−31.35, −12.09)	−23.17 (−32.39, −13.49)	0.048 (−0.016, 0.144)	−0.1
	GGT	0.011 (−0.004, 0.031)	0.265 (0.188, 0.343)	0.277 (0.194, 0.363)	0.042 (−0.016, 0.114)	0.2
	ALP	−0.001 (−0.004, 0.0008)	−0.030 (−0.065, 0.004)	−0.031 (−0.067, 0.004)	0.038 (−0.079, 0.348)	0
	US-based steatosis	0.004 (−0.001, 0.012)	0.053 (−0.008, 0.117)	0.058 (−0.004, 0.121)	0.071 (−0.194, 0.467)	0.1
	US-based NAFLD	0.003 (−0.001, 0.011)	0.246 (0.177, 0.309)	0.250 (0.181, 0.314)	0.013 (−0.005, 0.044)	0.1

1 *n* = 705. The table depicts results from mediation analysis where alcohol consumption was treated as the exposure; the outcomes were liver-related traits including CT-based fatty liver, liver enzymes (GGT and ALP), and US-based steatosis and NAFLD; and miRNAs were the mediators. ACME reflects the proportion of alcohol exposure on liver-related traits mediated through the miRNA of interest, whereas ADE reflects the direct effect of alcohol consumption on liver-related traits. Prop. Med. reflects the proportion mediated which cannot be calculated when the indirect and direct effects are in opposite directions, ρ at which ACME is 0, depicting how sensitive the tested model is to violation of unmeasured confounding. ACME, average causal mediation effect; ADE, average direct effect; ALP, alkaline phosphatase; CT, computed tomography; GGT, γ-glutamyl transferase; miR, microRNA; miRNA, microRNA; NAFLD, nonalcoholic fatty liver disease; US, ultrasound.

### MR

We investigated the causal relation between the alcohol-associated miR-193-5p and liver fat percentage, NAFLD, and liver enzymes (https://finngen.gitbook.io/documentation/) (51–53, 55, 56, 78). **Supplemental Table 4** presents the results of the MR analysis. There was no statistical evidence for a causal relation between alcohol-associated miRNAs and the liver-related traits tested.

### Liver expression and target genes of alcohol-associated miRNAs

Publicly available tools were utilized to assess the expression of alcohol-associated miRNAs across a wide range of tissues (**Supplemental Table 5**). Among these, miR-122-5p had the highest TSI of 0.97 (where a higher score indicates miRNA is expressed in a single tissue) (Supplemental Table 5). In addition, miR-122-5p and miR-4507 displayed the highest expression in the liver tissue, whereas miR-193b-3p showed the highest expression in muscle and miR-4507 in the stomach.

**Supplemental Table 6** shows potential target genes of the alcohol-associated miRNAs. Only miR-193b-3p and miR-122-5p had validated target genes by experimental methods as reported in miRTarBase (Supplemental Table 6) (71). By performing a literature review, we identified that several putative target genes of miR-193b-3p, miR-122-5p, and miR-3937 had been previously associated with alcohol-related traits (**Supplemental Table 7**). These included *FLI* and *SMAD3*, both putative targets of miR-193b-3p, which were previously identified in an EWAS on alcohol consumption (38). In addition, putative target genes of miR-122-5p (*XPO6* and *SLC7A11*) were identified in the same EWAS, along with *C7orf50*, a putative target gene of miR-3937 (Supplemental Table 7) (38). Furthermore, *DCLK2*, 1 of the miR-3937 putative target genes, was previously associated in a trans-ethnic genome-wide association analysis of Alcohol Use Disorder Identification Test (AUDIT)-Consumption (rs4423856, *P* = 1.48 × 10^−8^) (74). Also, an miR-122-5p putative target gene, *RAC1*, was previously associated with alcohol use during pregnancy (33, 79). *FOXP1*, another putative target gene of miR-122-5p, was previously reported in a transcriptome-wide association study on alcohol intake frequency (http://twas-hub.org/traits/) (80, 81).

**Supplemental Table 8** presents our biological processes overrepresentation analysis on the putative target genes of alcohol-associated miRNAs. The top biological process pathways were the following: biological regulation, biological process, and the transmembrane receptor protein serine/threonine kinase signaling pathway (Supplemental Table 8).

## Discussion

In this study, we investigated the link between plasma miRNA expression and alcohol consumption in a population-based setting. We identified plasma concentrations of 4 miRNAs to be significantly associated with alcohol consumption, including 3 miRNAs positively and 1 miRNA inversely associated. Among these, we observed a potential mediatory role of miR-122-5p and miR-3937 between alcohol consumption and liver-related traits. The identified miRNAs lay the groundwork for further investigation of miRNAs as potential mediators between modifiable lifestyle factors and disease risk.

miRNAs could modulate gene expression in response to external influences, such as lifestyle factors (e.g., smoking, alcohol consumption, and diet) (33). It has been shown that miRNA expression was altered after exposure to maternal alcohol consumption during human embryogenesis (82–84). Similarly, Lewohl et al. (23) have identified differential expression of 35 miRNAs in human postmortem brains between 14 alcoholics and 13 controls. However, most of the previous studies exploring the association between alcohol consumption and miRNA expression were performed on animal models (85–87). In addition, past research either has been conducted on a subset of miRNA or had relatively modest sample sizes (with the largest sample size reported *n* = 68) (33). Our study benefits from a greater statistical power to detect significant associations between miRNAs and alcohol consumption due to the larger sample size embedded in the population-based RS cohort. In addition, the RNA-sequencing method was used to measure a large number of miRNAs, enabling us to investigate a more comprehensive miRNA landscape (88).

The most prominent association with alcohol consumption was observed for miR-193b-3p. Previous studies have identified miR-193 as a regulator of *ALDH2* gene expression across different species (89), where the *ALDH2* gene encodes alcohol aldehyde dehydrogenase 2, a key enzyme in alcohol metabolism (90). This miRNA has several other putative target genes, including *FLI* and *SMAD3*, previously identified in an EWAS on alcohol consumption (38). The same study overlapped with other putative target genes of our newly identified alcohol miRNAs, including *XPO6* and *SLC7A11* of miR-122-5p and *C7orf50* of miR-3937 (38). In addition, miR-122-5p expression has been shown to increase with moderate ethanol consumption in healthy individuals (35). In line with this, 2 target genes of miR-122-5p were linked with alcohol consumption, including *RAC1* with alcohol use during pregnancy (33, 79) and *FOXP1* in a transcriptome-wide association study on alcohol intake frequency (http://twas-hub.org/traits/) (80, 81). *DLCK2* is a target gene of miR-3937, linked with the AUDIT (rs4423856, *P* = 1.48 × 10^−8^) (74). The last alcohol-associated miRNA identified in our study (miR-4507) was previously reported by Gardiner et al. (79) when comparing alcohol consumption with alcohol abstinence during pregnancy. Multiple target genes of alcohol-associated miRNAs were linked to alcohol consumption through other omics analyses (Supplemental Table 7) (33, 38, 74). In addition, most of the identified miRNAs were previously implicated in liver diseases, which is unsurprising because the liver is a primary organ for alcohol metabolism and detoxification (16, 91, 92). For instance, miR-193b-3p, miR-3937, and miR-122-5p were linked with fatty liver disease in the RS (48). In addition, miR-122-5p is firmly recognized as a liver-specific miRNA (93) with an undeniably established role in liver function and related diseases (93–95). These results corroborate well with the findings linking the newly identified miRNAs to alcohol consumption. When we explored alcohol consumption as a categoric exposure (nondrinkers, light drinkers, and heavy drinkers), despite the smaller sample size, the effect estimate for heavy drinkers was almost double than for light drinkers for the alcohol-associated miRNAs when comparing with the nondrinkers group (Table 2). In our sensitivity analysis, we identified that most of the alcohol-associated miRNAs had stronger effect estimates in men, perhaps due to the higher consumption of alcohol.

Our mediation analyses showed a potential mediatory role of miR-122-5p in the association of alcohol consumption and CT-based fatty liver disease, GGT, and US-based steatosis. Moreover, we observed a mediating effect of miR-3937 in the association between alcohol consumption and CT-based fatty liver and GGT. This may indicate a significant estimated indirect effect of alcohol consumption on liver function or disease that is mediated partly through miR-3937 and miR-122-5p. In addition, we did not find any statistical evidence for causality between alcohol-associated miRNAs and liver-related traits. However, we believe that these results might have been hampered by the lack of strong IVs, because we only found a single SNP as a valid IV. This warrants future studies to perform large-scale GWASs on a broad landscape of miRNAs, providing stronger IVs for estimating causal relations.

This study has strengths as well as limitations that should be considered when interpreting the results. The strengths of our study include the large sample size, availability of clinical outcomes, and using a new RNA-sequencing-based assay with high sensitivity. Yet, it is plausible that several limitations could have influenced the results presented. First, mediation analysis requires strong assumptions whose violations might lead to spurious results, such as no unmeasured confounding. In line with this, implementing mediation analysis in cross-sectional observational studies and notably in genomic studies is challenging and adds a layer of complexity (96). We implemented bias analyses to explore if the assumption of no unmeasured confounding held. Given the cross-sectional nature of the data used for the presented study, we cannot rule out reverse causality. In line with this, data on miRNAs, alcohol consumption, fatty liver, and liver enzymes were measured at the same time point, whereas US data were analyzed in a longitudinal setting. Although we adjusted for potential confounders, there might still be residual confounding due to the dynamic nature of epigenetic markers, which might partially explain some of the ρ values close to 0 we obtained from bias analyses within mediation analyses. Future analyses are warranted to replicate the findings from our study and explore these findings in a longitudinal setting. In addition, future studies are needed to explore the dynamic nature of epigenetic markers such as miRNAs and explore reverse causation, especially in the context of mediation analysis. Another source of bias might have occurred from the CT scan used for LA (97); however, we also included data on US-based measurements. In addition, the FibroScan is currently an often used method in the clinic to determine liver fat and fibrosis, whereas we used CT scans in the current study. Nevertheless, large cohort studies are more likely to use CT scans owing to their broad applications, making possible direct replication of our obtained results by other studies. Also, because miRNAs are tissue-specific, we might have missed important miRNAs in relevant tissue such as liver. However, the accessibility of plasma compared with other tissues provides a potential benefit for identified miRNAs to serve as indicators for alcohol exposure (98). In addition, we utilized the Tissue Atlas database (https://ccb-web.cs.uni-saarland.de/tissueatlas) (68, 69) in order to explore the expression of the alcohol-identified miRNAs across a wide range of tissues.

In addition, it is important to address the potential limitation coming from the data on alcohol consumption, because they were collected by home-administered interviews and not by FFQs or other validated self-reports, such as AUDIT (https://auditscreen.org/) (99). Although the FFQs are more detailed and AUDIT is more effective in screening individuals with unhealthy alcohol use, we did not have data derived from FFQ or AUDIT on this wave of participants. In addition, participants might have underestimated their true alcohol consumption owing to social desirability bias. Finally, it is important to acknowledge the potential risk of introducing type I error in our additional analysis because we did not correct for multiple testing. Given the nature of high correlation of omics data, we believe the potential risk of introducing type I error in our additional analysis is accounted for, to a certain extent. Further studies are needed to replicate our findings using larger sample sizes and longer follow-up times as well as to experimentally confirm the role of identified miRNAs in molecular pathways underlying alcohol-related diseases.

In conclusion, we showed in a population-based setting that alcohol consumption was associated with plasma concentrations of 4 miRNAs, 2 of which showed a potential mediatory role on liver-related traits. This might provide a better understanding of the mechanism of action involved between alcohol consumption and alterations in gene expression in alcohol-related diseases.

## Supplementary Material

nxac216_Supplemental_File

## Data Availability

Data used in this article will not be made available because of the confidential nature of the data collected; analytic code will be made available upon reasonable request to the corresponding author.

## Notes

The authors reported no funding received for this study.

Author disclosures: the authors report no conflicts of interest.

Supplemental Tables 1–8 are available from the “Supplementary data” link in the online posting of the article and from the same link in the online table of contents at https://academic.oup.com/jn/.

YA and SCEM contributed equally to this work.

Abbreviations used: ACME, average causal mediation effect; ALP, alkaline phosphatase; AUDIT, Alcohol Use Disorder Identification Test; CPM, counts per million; CT, computed tomography; EWAS, epigenome-wide association study; GGT, γ-glutamyl transferase; GWAS, genome-wide association study; IV, instrumental variable; LA, liver attenuation; LLOQ, lower limit of quantification; miR, microRNA; miRNA, microRNA; MR, Mendelian randomization; NAFLD, nonalcoholic fatty liver disease; ROI, region of interest; RS, Rotterdam Study; TSI, tissue specificity index; US, ultrasound; WTA, Whole Transcriptome Assay.
