# Supplementary material for: Plasma MicroRNA Signature of Alcohol Consumption: The Rotterdam Study
Source: J Nutr. 2022 Sep 20;152(12):2677–88. doi: 10.1093/jn/nxac216 (PMC9839997; doi:10.1093/jn/nxac216)
Supplement: nxac216_Supplemental_File [file nxac216_supplemental_file.docx]

**Plasma microRNA signature of alcohol consumption: the Rotterdam Study**

**Irma Karabegović et al.**

**Online Supplemental data**

**Article:** Plasma microRNA signature of alcohol consumption: the Rotterdam Study

**Authors:** Irma Karabegović, Yasir Abozaid, Silvana C.E. Maas, Jeremy Labrecque, Daniel Bos, Robert J. De Knegt, M. Arfan Ikram, Trudy Voortman, Mohsen Ghanbari

**Supplemental Tables**

**Supplemental Table S1.** Baseline characteristics of the study population subset used for mediation analysis

**Supplemental Table S2.** Sex-stratified sensitivity analysis for alcohol-associated miRNAs

**Supplemental Table S3.** Mediation analysis with interaction terms between alcohol consumption and tested mediators

**Supplemental Table S4.** Mendelian randomization analysis exploring the causal role between alcohol-associated miRNAs and liver-related traits

**Supplemental Table S5.** The expression of six alcohol-associated miRNAs in the liver tissue utilizing TissueAtlas database

**Supplemental Table S6.** List of putative target genes of six alcohol-associated miRNAs retrieved through miRNA-target prediction web tools

**Supplemental Table S7.** miRNA putative target genes previously linked with alcohol consumption

**Supplemental Table S8.** Gene ontology analyses identifying the potential biological processes of the miRNA predicted target genes

**Supplemental Table S1. Baseline characteristics of the study population subset used for mediation analysis**

| Variable | N=705 |
| --- | --- |
| Age (years) | 67.58 (±5.6) |
| Female sex (%) | 380 (53.9) |
| Smoking |  |
| Current (%) | 88 (12.5) |
| Former (%) | 398 (56.5) |
| Never (%) | 219 (31.1) |
| Alcohol (glasses/day) | 0.10 (0.14 – 2.04) |
| Non-drinkers (%)^a^ | 87 (12.3) |
| Light-drinkers (%)^b^ | 355 (50.4) |
| Heavy-drinkers (%)^c^ | 263 (37.3) |
| CT-based HU liver attenuation | 61.9 (55.8 - 65.7) |
| GGT (U/L) | 25.0 (18.0 - 40.0) |
| ALP (U/L) | 76.0 (66.0 - 90.0) |
| Hepatic steatosis (%) | 252 (35.7) |
| NAFLD (%) | 331 (47.0) |

Characteristics of the subset of participants who had data available on miRNA levels, alcohol consumption, and liver-related traits used for mediation analyses. Variables are reported in mean (standard deviation (SD)) for continuous data and numbers (percentages) for categorical data, apart from alcohol, liver attenuation, and liver enzymes (GGT and ALP) reported in median and IQR, due to the distribution of the variables. Alcohol categories were defined as following: ^a^non-drinkers: glasses/day=0, ^b^light drinkers: 0<-≤2 glasses/day in men and 0<-≤1 glasses/day in women, and ^c^heavy drinkers: >2 glasses/day in men and >1 glasses/day in women.

**Supplemental Table S2. Sex-stratified sensitivity analysis for alcohol-associated miRNAs**

| miRNA | Combined (n=1933) | | | Women (n=1098) | | | Men (n=835) | | | |
| --- | --- | --- | --- | --- | --- | --- | --- | --- | --- | --- |
|  | β | SE | P-value | β | SE | P-value | β | SE | P-value |  |
| miR-193b-3p | 0.087 | 0.020 | 2.90×10^-5^ | 0.055 | 0.030 | 6×10^-2^ | 0.097 | 0.028 | 7.24×10^-4^ |  |
| miR-122-5p | 0.151 | 0.037 | 4.31×10^-5^ | 0.045 | 0.053 | 3.99×10^-1^ | 0.220 | 0.051 | 1.96×10^-5^ |  |
| miR-3937 | 0.145 | 0.036 | 5.71×10^-5^ | 0.138 | 0.053 | 9.01×10^-3^ | 0.151 | 0.049 | 2.33×10^-3^ |  |
| miR-4507 | -0.110 | 0.027 | 8.36×10^-5^ | -0.144 | 0.043 | 9.30×10^-4^ | -0.079 | 0.035 | 2.61×10^-2^ |  |

Results from the sex-stratified analysis where alcohol exposure is a continuous variable transformed to (log(glass/day)+1 ) and treated as main exposure, while outcomes are alcohol-associated miRNAs adjusted for age, sex, cohort, BMI, and smoking status. Stratified analyses were performed on women (left side) and men (right side) separately and were not adjusted for sex. Effect size estimates are beta coefficients from linear regression analysis. Abbreviations: β:beta coefficient, SE: standard error

**Supplemental Table S3. Mediation analysis with interaction terms between alcohol consumption and tested mediators (N=705)**

| miRNAs | Liver-related traits (N=705) | ACME (95%CI) | ADE  (95%CI) | Total effect (95%CI) | *Prop. Med. (95%CI) | $\boldsymbol{\rho}$ at which ACME is 0 |
| --- | --- | --- | --- | --- | --- | --- |
| miR-3937 | CT - based fatty liver | 1.729 (0.027; 3.842) | -24.90 (-34.74; -15.21) | -23.17 (-32.85; -13.26) | -0.074 (-0.227;0.0002) | 0.1 |
|  | GGT | -0.008 (-0.021; -0.000) | 0.286 (0.203;0.368) | 0.277 (0.194; 0.359) | -0.030 (-0.080;-0.000) | -0.1 |
|  | ALP | -0.002 (-0.007; 0.0005) | -0.029  (-0.067; 0.007) | -0.032 (-0.069; 0.005) | 0.082 (-0.180; 0.624) | -0.1 |
|  | US - based steatosis | -0.006 (-0.016; 0.0001) | 0.063  (0.001; 0.121) | 0.057 (-0.005; 0.114) | -0.105 (-1.137;0.534) | NA ** |
|  | US - based NAFLD | -0.004 ( -0.013; 0.001) | 0.255  (0.192; 0.321) | 0.251 (0.186; 0.318) | -0.018 (-0.058; 0.005) | NA ** |
| miR-122-5p | CT - based fatty liver | -1.256 ( -2.914; -0.088) | -21.538  (-32.011; -11.193) | -22.794  (-33.475; -12.301) | 0.055 (-0.004; 0.154) | -0.1 |
|  | GGT | 0.034 (0.008; 0.068) | 0.237 (0.168; 0.308) | 0.272 (0.195; 0.348) | 0.128 (0.032; 0.240) | 0.4 |
|  | ALP | 0.002 (-0.0009; 0.006) | -0.029  (-0.067; 0.005) | -0.027  (-0.065; 0.006) | -0.085( -0.877; 0.356) | 0 |
|  | US - based steatosis | 0.008 (0.0007; 0.020) | 0.047 (-0.013; 0.110) | 0.055 (-0.002; 0.119) | 0.147  (-0.598; 1.179) | NA ** |
|  | US - based NAFLD | 0.005 (-0.0005; 0.015) | 0.244 (0.180; 0.310) | 0.250 (0.186; 0.314) | 0.022 (-0.002; 0.066) | NA ** |
| miR-193b-3p | CT - based fatty liver | -1.338 (-3.392; 0.431) | -22.59 (-31.34; -12.06) | -23.936 (-32.428; -13.477) | 0.055 (-0.021; 0.159) | -0.1 |
|  | GGT | 0.010 (-0.003; 0.025) | 0.265 (0.180; 0.339) | 0.276 (0.188; 0.350) | 0.037 (-0.014; 0.097) | 0.1 |
|  | ALP | -0.0003 (-0.003; 0.001) | -0.030 (-0.064; 0.006) | -0.030 (-0.064; .006) | 0.013 (-0.140; 0.258) | 0 |
|  | US - based steatosis | 0.004 (-0.001; 0.013) | 0.054  (-0.013; 0.109) | 0.058 (-0.010; 0.114) | 0.074 (-0.157; 0.644) | NA ** |
|  | US - based NAFLD | 0.003 (-0.001; 0.011) | 0.247 (0.187; 0.311) | 0.250 (0.190; 0.314) | 0.013 (-0.006; 0.048) | NA ** |

The table depicts results from mediation analysis with interaction effects between exposure (alcohol consumption) and mediators (miR-3937, miR-122-5p, and miR-193b-3p). ACME reflects the proportion of alcohol exposure on liver-related traits mediated through miRNA of interest, while ADE reflects the direct effect of alcohol consumption on liver-related traits. *Prop. Med. reflects proportion mediated - which cannot be calculated when the indirect and direct effects are in opposite directions, ρ at which ACME is 0, depicting how sensitive the tested model is to violating of unmeasured confounding, NA** bias analysis is not available for binary outcome with interaction terms. Abbreviations: β: beta coefficient, SE: standard error, ACME: Average Causal Mediation Effect, ADE: Average Direct Effect.

**Supplemental Table S4.** Mendelian randomization analysis exploring the causal role between alcohol-associated miRNAs and liver-related traits

| Method | Exposure | Outcome | β | SE | P-value |
| --- | --- | --- | --- | --- | --- |
| Wald Ratio | miR-193-5p | Liver fat percentage | -0.028 | 0.039 | 0.463 |
|  |  | NAFLD | 0.232 | 0.247 | 0.347 |
|  |  | Liver enzymes | -0.002 | 0.001 | 0.227 |

Results are presented for Wald ratio method MR, as a single SNP as an instrumental variable (IV) was available for all of the alcohol-associated miRNAs tested. Exposure variable IVs (cis-miR-eQTLs) were extracted using Rotterdam Study and publicly available resources (1-3). Outcome variable IVs were extracted using publicly available resources (<https://finngen.gitbook.io/documentation/>) (4, 5).

**Supplemental Table S5. The expression of six alcohol-associated miRNAs in the liver tissue utilizing TissueAtlas database**

| miRNA | Tissue specificity index | Liver tissue |
| --- | --- | --- |
| miR-193b-3p | 0.86 | 62.19 |
| miR-122-5p | 0.97 | 4468.79 |
| miR-3937 | 0.58 | 316.39 |
| miR-4507 | 0.62 | 6462.63 |

The table shows the expression levels of six alcohol-associated miRNAs in the liver tissue as obtained from the TissueAtlas database: Human miRNA tissue atlas database (<https://ccb-web.cs.uni-saarland.de/tissueatlas>) (6, 7). Tissue specificity index (range 0 to 1) refers to the level of expression of given miRNA across different tissue. A lower score corresponds that miRNA is expressed in multiple tissues, while a higher score means miRNA is expressed in a single tissue. Abbreviations: miR- microRNA; NA- not available.

**Supplemental Table S6. List of putative target genes of six alcohol-associated miRNAs retrieved through miRNA-target prediction web tools**

| miRNA ID | Putative target genes |
| --- | --- |
| miR-193b-3p | AFF4, AP2M1, CBX7, DCAF7, DOK6, ERBB4, FAM84A, FHDC1, FLI1, GPR20, HHAT, HOXD13, IL17RD, KRAS, LYRM2, PTPN9, RTN4IP1, SIRT7, SLC10A6, SLC16A6, SLC39A5, SRSF2, ST6GALNAC5, TAF7L, TANGO2, VN1R1, ZBTB5, ZNF510, CCND1, PLAU, MCL1, ETS1, MAX, NF1, SMAD3 |
| miR-122-5p | ALDOA, CCAR1, CLIC4, CTDNEP1, MASP1, PLEKHB2, RFXAP, SLC52A2, ZNF827, CYP7A1, WNT1, IGF1R, SRF, RAC1, RHOA, PRKRA, CCNG1, GTF2B, GYS1, ANK2, NFATC2IP, ENTPD4, ANXA11, RAB6B, RAB11FIP1, FOXP1, MECP2, NCAM1, UBAP2, TBX19, AACS, DUSP2, ATP1A2, MAPK11, FUNDC2, AKT3, TPD52L2, GALNT10, G6PC3, P3M2, SLC7A1, XPO6, FOXJ3, SLC7A11, TRIB1, EGLN3, NUMBL, ADAM17, Tgfbr1, Sbk1, Hist1h1c, Ddc, Rell1, Bach1, Apob, P4ha1, Ccng1, Slc7a1, Gys1, Slc35a4, Hfe2, Tmed3, Bckdk, Aldoa, Ndrg3, Cd320, Sox4, Prom1, Cs, Igf2, Il1b, Dbp, Cyp2b13, Cxcl13, Smarcd1, Rcan1, Gde1, DSTYK, FAM117B, BCL2L2, PRKAB1, Ccrn4l, Ccl2, ADAM10, Klf6, ACVR1C, Alpl, Per1, Jun, Ctgf, Afp, B2m, Csf3r,  PTPN1, NT5C3A, P4HA1, ZNF395, SOCS1, Hamp, Tfr2, Smad4, Smad7, Bmpr1a, Hba-a1, Hmbs, Mir17, Alas2, Mir451, Tfrc, Socs2, Slc35g1, Camk2b, Irf6, Rbl2, Hfe, Ccnd1, HMOX1, BCL2L1, PKM, BAX, CDK4, Pkm, Tfdp2, E2f1, Cux1, MEF2D, TGFB1, AXL, NOD2, FUT8 |
| miR-3937 | C7orf50, DCAF12L2, DCLK2, GRM4, KRT82, TAP11-1, M1AP, NPW |
| miR-4507 | ADAM19, C16orf95, CHTF8, DENND1A, GHITM, GIPC3, HCN1, KPNA7, LMLN, MRPL20, SMIM10, TAPBPL, TBC1D3G, TBC1D3H, THBS4, TRAPPC2 |

Putative target genes were obtained through three commonly used miRNA target genes prediction databases: TargetScan (8), miRTarBase (9), and miRDB (10). In this table, genes that passed the thresholds explained in the methods section of the manuscript and that were reported in at least two databases used for gene ontology analysis are shown.

**Supplemental Table S7.** **miRNA putative target genes previously linked with alcohol consumption**

| miRNA | Putative target genes | GWAS (11) | EWAS (12) | TWAS  (<http://twas-hub.org/traits/>) (13, 14) | Review (15) |
| --- | --- | --- | --- | --- | --- |
| miR-193b-3p | FLI, SMAD3 | *-* | FLI, SMAD3 | *-* | *-* |
| miR-122-5p | FOXP1, XPO6, SLC7A11, RAC1 | *-* | XPO6, SLC7A11 | FOXP1 | RAC1 |
| miR-3937 | C7orf50, DCLK2 | DCLK2 | C7orf50 | *-* | *-* |
| miR-4507 | *-* | *-* | *-* | *-* | *-* |

A literature search of alcohol-associated miRNA predicted target genes, in order to assess if they were previously implicated in relation to alcohol consumption with other omics data.

**Supplemental Table S8. Gene ontology analyses identifying potential biological processes of the miRNA predicted target genes**

| GO Enrichment Analysis- biological process | Raw P value | FDR |
| --- | --- | --- |
| biological regulation | 5.04×10^-9^ | 1.12×10^-5^ |
| Unclassified | 8.92×10^-9^ | 9.90×10^-6^ |
| Biological process | 8.92×10^-9^ | 6.60×10^-6^ |
| transmembrane receptor protein serine/threonine kinase signaling pathway | 2.66×10^-7^ | 1.47×10^-4^ |
| regulation of cellular process | 5.31×10^-7^ | 2.36×10^-4^ |
| regulation of biological process | 5.41×10^-7^ | 2.00×10^-4^ |
| cellular process | 1.64×10^-6^ | 5.19×10^-4^ |
| cell surface receptor signaling pathway | 2.08×10^-6^ | 5.76×10^-4^ |
| BMP signaling pathway | 2.21×10^-6^ | 5.46×10^-4^ |
| transcription by RNA polymerase II | 2.37×10^-6^ | 5.26×10^-4^ |
| cellular response to BMP stimulus | 2.44×10^-6^ | 4.92×10^-4^ |
| response to BMP | 2.44×10^-6^ | 4.51×10^-4^ |
| enzyme linked receptor protein signaling pathway | 3.37×10^-6^ | 5.75×10^-4^ |
| response to growth factor | 5.14×10^-6^ | 8.15×10^-4^ |
| cellular response to growth factor stimulus | 5.14×10^-6^ | 7.61×10^-4^ |
| phosphorylation | 1.21×10^-5^ | 1.69×10^-3^ |
| regulation of transcription by RNA polymerase II | 1.25×10^-5^ | 1.64×10^-3^ |
| cellular response to transforming growth factor beta stimulus | 1.29×10^-5^ | 1.60×10^-3^ |
| response to transforming growth factor beta | 1.29×10^-5^ | 1.51×10^-3^ |
| transforming growth factor beta receptor signaling pathway | 1.29×10^-5^ | 1.44×10^-3^ |
| metabolic process | 1.77×10^-5^ | 1.87×10^-3^ |
| extrinsic apoptotic signaling pathway in absence of ligand | 2.73×10^-5^ | 2.75×10^-3^ |
| intrinsic apoptotic signaling pathway in response to DNA damage | 3.31×10^-5^ | 3.19×10^-3^ |
| nucleic acid-templated transcription | 4.02×10^-5^ | 3.72×10^-3^ |
| transcription, DNA-templated | 4.02×10^-5^ | 3.57×10^-3^ |
| regulation of metabolic process | 4.09×10^-5^ | 3.49×10^-3^ |
| RNA biosynthetic process | 4.18×10^-5^ | 3.44×10^-3^ |
| organic cyclic compound biosynthetic process | 5.09×10^-5^ | 4.03×10^-3^ |
| signal transduction | 5.96×10^-5^ | 4.56×10^-3^ |
| cellular response to organic substance | 7.77×10^-5^ | 5.75×10^-3^ |

We performed a gene ontology biological statistical overrepresentation test using the web tool PANTHER (<http://www.pantherdb.org/>)(16). The biological processes are sorted based on the ascending P value. Raw P value is determined by Fisher’s exact test, representing the probability that the number of genes observed in this category occurred randomly, while the false discovery rate (FDR) was computed via the Benjamin-Hochberg procedure.

**References**

1. Akiyama S, Higaki S, Ochiya T, Ozaki K, Niida S, Shigemizu D. JAMIR-eQTL: Japanese genome-wide identification of microRNA expression quantitative trait loci across dementia types. Database (Oxford). 2021;2021(2021).

2. Nikpay M, Beehler K, Valsesia A, Hager J, Harper ME, Dent R, et al. Genome-wide identification of circulating-miRNA expression quantitative trait loci reveals the role of several miRNAs in the regulation of cardiometabolic phenotypes. Cardiovasc Res. 2019;115(11):1629-45.

3. Huan T, Rong J, Liu C, Zhang X, Tanriverdi K, Joehanes R, et al. Genome-wide identification of microRNA expression quantitative trait loci. Nat Commun. 2015;6:6601.

4. Liu Y, Basty N, Whitcher B, Bell JD, Sorokin EP, van Bruggen N, et al. Genetic architecture of 11 organ traits derived from abdominal MRI using deep learning. Elife. 2021;10.

5. Pazoki R, Vujkovic M, Elliott J, Evangelou E, Gill D, Ghanbari M, et al. Genetic analysis in European ancestry individuals identifies 517 loci associated with liver enzymes. Nat Commun. 2021;12(1):2579.

6. Ludwig N, Leidinger P, Becker K, Backes C, Fehlmann T, Pallasch C, et al. Distribution of miRNA expression across human tissues. Nucleic Acids Res. 2016;44(8):3865-77.

7. University CfCB-S. Tissue Atlas: Chair for Clinical Bioinformatics - Saarland University; May 2016 [updated July 2022. Available from: <https://ccb-web.cs.uni-saarland.de/tissueatlas>.

8. Agarwal V, Bell GW, Nam JW, Bartel DP. Predicting effective microRNA target sites in mammalian mRNAs. Elife. 2015;4.

9. Huang HY, Lin YC, Li J, Huang KY, Shrestha S, Hong HC, et al. miRTarBase 2020: updates to the experimentally validated microRNA-target interaction database. Nucleic Acids Res. 2020;48(D1):D148-D54.

10. Chen YH, Wang XW. miRDB: an online database for prediction of functional microRNA targets. Nucleic Acids Research. 2020;48(D1):D127-D31.

11. Kranzler HR, Zhou H, Kember RL, Smith RV, Justice AC, Damrauer S, et al. Genome-wide association study of alcohol consumption and use disorder in 274,424 individuals from multiple populations. Nat Commun. 2019;10.

12. Liu C, Marioni RE, Hedman AK, Pfeiffer L, Tsai PC, Reynolds LM, et al. A DNA methylation biomarker of alcohol consumption. Mol Psychiatry. 2018;23(2):422-33.

13. School GLatD-FCIaHM. TWAS hub: Gusev Lab at the Dana-Farber Cancer Institute and Harvard Medical School; 06/10/2018 [updated 09/19/2018 Available from: <http://twas-hub.org/traits/>.

14. Gusev A, Ko A, Shi H, Bhatia G, Chung W, Penninx BW, et al. Integrative approaches for large-scale transcriptome-wide association studies. Nat Genet. 2016;48(3):245-52.

15. Panico A, Tumolo MR, Leo CG, Donno A, Grassi T, Bagordo F, et al. The influence of lifestyle factors on miRNA expression and signal pathways: a review. Epigenomics. 2021;13(2):145-64.

16. Project GOPA. PANTHER: Gene Ontology Phylogenetic Annotation Project; [updated 22-02-2022.
